# Looking back to move forward - lessons from clinical trial activity and growth in rural, regional and remote Australia: a scoping review and evidence map

**DOI:** 10.3389/fmed.2026.1977490

**Published:** 2026-10-29

**Authors:** Jenna Graffini, Katri Malinen, Michelle A. Krahe, Karen Carlisle, Steven M. McPhail, Sarah Larkins

**Affiliations:** 1 College of Medicine and Dentistry, James Cook University, Townsville, QLD, Australia; 2 Australian Institute of Tropical Health and Medicine, James Cook University, Townsville, QLD, Australia; 3 Townsville Institute of Health Research and Innovation, Townsville University Hospital, Townsville Hospital and Health Service, Townsville, QLD, Australia; 4 Australian Centre for Health Services Innovation and Centre for Healthcare Transformation, School of Public Health and Social Work, Faculty of Health, Queensland University of Technology, Brisbane, QLD, Australia

**Keywords:** capacity building, clinical trial, clinical trial activity, participant recruitment, research involving first nations Australians, rural Australia

## Abstract

**Background:**

About one-third of Australians in rural, regional, and remote areas have limited access to clinical trials, leading to increased 2019 government funding to improve access.

**Objectives:**

To review experiences of conducting clinical trials in these regions, describe efforts to develop activity, and identify gaps for future research.

**Eligibility criteria:**

English-language peer-reviewed literature reporting on clinical trial activity, development, implementation, or lessons learned in rural, regional, and remote Australia. We excluded protocols, non-peer-reviewed literature, urban-focused studies, and outcome-focused clinical trial reports.

**Sources of evidence:**

A systematic search of Scopus (MEDLINE), Informit Health Collection, and CINAHL electronic databases was conducted from inception to September 2024.

**Charting methods:**

Data were extracted using a bespoke tool iteratively developed from previous literature and piloting.

**Results:**

Of 466 records identified, 435 were screened, 84 underwent full-text review, and 24 were included. Eighteen were empirical with diverse research methodologies, and six were non-empirical. A third of publications (*n* = 8) reported on trials conducted in Victoria, and over half (*n* = 13) focused on oncology clinical trials. A hybrid deductive-inductive thematic analysis identified four key themes: 1) perceptions of clinical trials, 2) clinical trial activity and capability, 3) participant recruitment, and 4) clinical trials involving First Nations Australians. Clinicians and patients generally viewed clinical trials as important; however, limited clinical trial capability and maturity constrained local health services. Studies reporting economic analysis suggested that conducting clinical trials closer to participants could reduce costs for those who would otherwise need to travel to take part. Evidence indicates that First Nations Australians have been systematically excluded from clinical trials due to culturally inappropriate study designs.

**Conclusion:**

While trials are viewed positively, more research is needed to explore capability-building approaches that strengthen recruitment, minimise research costs, and ensure culturally safe approaches that support meaningful participation by First Nations Australians.

## 1 Introduction

Approximately 30% of Australians live in rural, regional or remote (RRR) areas and experience poorer health outcomes and healthcare access compared to their metropolitan counterparts (1). In this review, we define RRR as non-metropolitan areas, and it is categorised in Australia in two ways: the Accessibility/Remoteness Index of Australia (ARIA+) (2), which scores are grouped into five remoteness areas (major cities, inner regional, outer regional, remote, very remote) (3), and the Modified Monash Model (MMM) (4) which consists of seven categories (metropolitan, regional, large rural towns, medium rural towns, small rural towns, remote communities, very remote communities). Due to geographical remoteness, Australians living in RRR areas have reduced access to health care services for screening and treatment due to limited local infrastructure and workforce capacity, as well as long travel distances to specialist services (5). As a result, RRR Australians, particularly those with complex health conditions or cancer, may experience inadequate care such as delayed diagnosis and specialist treatment (6) and limited access to options such as clinical trials (7). Clinical trials, defined as a study that prospectively assigns consenting participants to a health intervention to evaluate the effects on health outcomes (8), can offer patients experimental treatments or diagnostic technologies that are otherwise unaffordable or not available in standard clinical practice (7). In oncology, offering patients a clinical trial, if available, is considered by the National Comprehensive Cancer Network as best practice (9). Clinical trials conducted within healthcare services offer numerous benefits, including increased participant knowledge of their health condition, enhanced quality of patient care in accordance with rigorous and evidence-based research protocols, and the advancement of healthcare translation through active engagement in research (7, 10). These benefits are not limited to oncology trials (10) and could potentially benefit RRR health services and their patients across a range of health disciplines. Furthermore, RRR Australia has some of the highest proportions of First Nations Australians (1). In this review, First Nations Australians refers to Aboriginal and Torres Strait Islander peoples, the First Peoples and original custodians of Australia. First Nations Australians are significantly under-represented in clinical trials despite experiencing poorer health outcomes compared to non-Indigenous people (11). Therefore, improving equity of access to clinical trials is important if Australia is serious about making meaningful progress in addressing the health disparity faced by people living in RRR areas.

In late 2024, our group published the first original research exploring the perceptions and experiences of health service staff, clinician researchers, and industry stakeholders about conducting clinical trials in RRR Australia before the implementation of the Australian Teletrial Program (12). This program was one of three programs funded by the $124.4 million Medical Research Future Fund 2019 RRR Clinical Trial Enabling Infrastructure Grant, the largest single investment made by the Australian Government to develop clinical trial capability in RRR Australia (13). We found universal challenges facing the clinical trial landscape in both metropolitan and RRR health services. There was enthusiasm among clinicians to offer their RRR patients clinical trials; however, a lack of a local skilled clinical trial workforce, investment, and infrastructure, and competing clinical priorities provided unique challenges to RRR health services. While these challenges are not unique to RRR Australia (14), Australian health services have an opportunity to lift their clinical trial standards and harmonise processes to improve equity of access to clinical trials. This ambitious task requires consideration of the past work up to this point to inform the future strategies and research in this space, and so the question was raised: what work had been done in Australia before September 2024 to enhance clinical trial activity in RRR areas, and what can we learn about the past to inform future direction? We defined the following objectives to guide the scoping review:

1. To identify and describe the experiences and lessons from conducting clinical trials in RRR regions of Australia.
2. To identify and describe how and where efforts to develop clinical trial activity in RRR regions of Australia have been directed.
3. To identify gaps and opportunities for further research to support the growth and sustainability of clinical trials in RRR Australia.

## 2 Methods

We conducted a scoping review of peer-reviewed publications exploring human clinical trial activity, capacity and capability within healthcare settings in RRR Australia. Search processes were conducted in accordance with the JBI methodology for scoping reviews (15) and reported in line with the PRISMA Extension for Scoping Reviews (16) (see Supplementary File 1). A review protocol was developed *a priori* to guide this review; however, it was not formally published, as protocol publication is recommended but not required for scoping reviews.

### 2.1 Eligibility criteria

The primary focus of this review was to include English-language, peer-reviewed research publications reporting on health-related clinical trials involving humans in RRR Australia (Table 1). The 2019 MRFF clinical trial infrastructure grant was awarded in 2020, with operational components of awarded programs commencing in 2021. An anticipated three-year publication lag (17) was incorporated into the search strategy to capture studies that had commenced up to 2021. The review included studies published from inception up to September 2024. Publications with diverse study designs (qualitative, quantitative and mixed-method) using both primary and/or secondary data were included to capture a wide range of perspectives (18). Publications were included if they explicitly reported on concepts relating to the conduct and development of clinical trial activity [e.g., barriers and enablers, lessons, clinical trial implementation outcomes (19)] in the Australian RRR context. For this reason, we excluded literature reviews, conference abstracts, and clinical trial protocols. In keeping with a scoping review's evidence-synthesising methodology (20), non-empirical publications were included (18) if their aims were exploring or describing the application of implementation concepts or frameworks in the RRR clinical trial context. We excluded general opinion or commentary papers not based on a specific implementation experience, program, initiative, or clinical trial context.

**Table 1:** Scoping review eligibility criteria for clinical trial activity and capacity in rural, regional, and remote Australia.

Criterion	Inclusion Criteria	Exclusion Criteria
Publication type	• Peer-reviewed publications	• Protocols • Opinions or commentary • Literature reviews • Conference reports or abstracts • Grey literature
Population	• Clinical trials involving patients of all ages, ages and ethnicities • People living in RRR Australia	• Clinical trials not involving patients • Studies not involving or about people living in RRR Australia
Concept	Studies must explicitly report on one or more of the below concepts: • Clinical trial activity, capacity or capability • Barriers, enablers or lessons learned in conducting clinical trials • Clinical trial implementation outcomes (acceptability, adoption, appropriateness, feasibility, fidelity, cost, penetration, sustainability)	Studies that primarily report on: • Clinical research capability, activity, capacity and not clinical trials • Clinical trial primary outcomes • Clinical trial protocols
Context	• Conducted in Australia • Clinical setting (Acute/Hospital, Primary Health Care, Community Health, Allied Health) • Rural, regional and remote setting (as reported in the study)	• Conducted in countries other than Australia • Clinical trials unrelated to health disciplines (e.g., school education, law/business, sociology, environmental health, public health: programs targeting a population) • Studies conducted exclusively in metropolitan settings.

### 2.2 Search

The search strategy was developed in consultation with a university librarian experienced in systematic and scoping review methods and focused on three key concepts: clinical trial capability, Australia and RRR. Relevant databases were identified, and preliminary searches were conducted to identify keywords, controlled vocabulary terms, and search operations (including Boolean operators and truncations). The search syntax was then refined for each database. The final search was conducted on the 24th September 2024 in CINAHL, Informit Health Collection and Scopus (MEDLINE) databases. The final search terms and strategy can be found in Supplementary File 2.

### 2.3 Selection of sources of evidence

Duplicate records were removed before screening. Two blinded authors (JG and KM) conducted the screening and full-text review using Rayyan (21), an online platform designed to facilitate the organisation of the systematic screening and selection process. A third reviewer (KC) was involved to resolve conflicts when needed.

### 2.4 Data charting process and data items

A bespoke data collection tool was developed iteratively, where initial fields were identified from previous work (12) and input was provided by researchers with experience in clinical trials and health systems research. The tool was further refined during pilot testing, and further data extraction, to ensure it was feasible to use, and variables were relevant and comprehensive to answer the review questions. The final tool captured four categories of information 1) study setting (state/territory, RRR+/- metro, health setting), 2) clinical trial of interest (health discipline, scope, sponsor); 3) study details (empirical status, methodology, primary/secondary data, data collection tools, source of data, description of outcome or concept); 4) quality appraisal rating. Publications were classified as empirical if they involved systematic collection, analysis, or synthesis of data to generate findings. Publications were classified as non-empirical if they primarily described, reflected on, or discussed clinical trial activities, implementation, lessons learned, policy, or program development without systematic analysis to generate findings. Collecting this information allowed us to understand and compare how the contextual factors may have influenced reported outcomes. The primary author (JG) extracted information from the publications, and the process was reviewed with co-researchers (KM, MK, SL, SM, KC).

### 2.5 Critical appraisal of individual sources of evidence

Quality appraisal of empirical studies was completed by JG using the Quality Assessment for Diverse Studies (QuADS) tool (22). While the QuADS does not provide a formal rating scale, the author developed a three-tier scoring system to present results as a star rating: one star represents the lowest quality score (0–13), two stars indicate a mid-range score (14–26), and three stars is the highest quality category (27–39).

### 2.6 Synthesis of results

The key outcomes or concepts of each publication were reviewed for emerging themes through a clinical trial landscape lens with a specific focus on feasibility and cultural considerations as conceptualised by several other authors (12, 23, 24). As a result, we applied a hybrid deductive-inductive thematic approach (25) informed by these predefined clinical trial feasibility domains, to synthesise the results. Studies were initially deductively grouped according to these clinical trial feasibility domains. Where the domains were not exhaustive to include all studies, new domains were developed. Within each domain, an inductive approach was used (26) to explore emerging patterns, concepts or insights from the study findings.

Data extracted from the studies were mapped to identify, organise, and visualise study characteristics and outcomes related to clinical trials in RRR Australia, and where applicable for non-empirical studies. The empirical studies were visually mapped by clinical focus, publication year, study type, remoteness, location, and setting. Additionally, an interactive visualisation was created using Kumu.io (27). It presents studies according to theme, with filters available for key study characteristics.

## 3 Results

### 3.1 Selection of sources of evidence

In total, 466 publications were identified by database searching (Figure 1). After removing duplicates, 435 publications were screened for relevance, with 351 non-relevant publications excluded, and 84 were assessed against the eligibility criteria (Table 1). In total, 24 publications were included in the review (Table 2).

**Figure 1:**
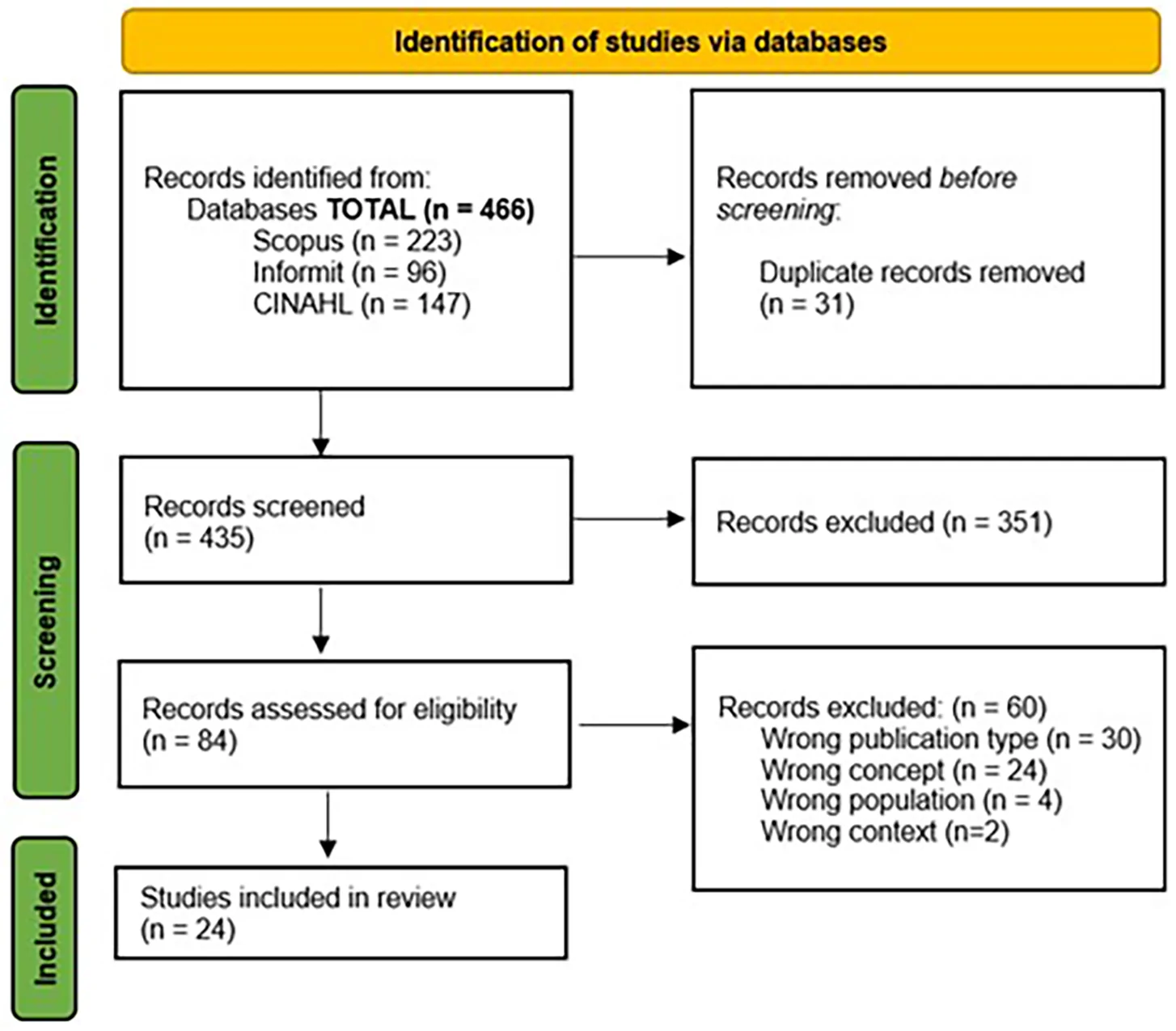
Flowchart of PRISMA screening process.

**Table 2:** Included publications that reported concepts relating to the conduct and development of clinical trial activity in RRR Australia.

First Author (ref)	Year	Study setting			Clinical trial of interest			Study details						Quality Rating*
		State or Territory	Geographic Setting	Health Setting	Health Discipline	Intervention type	Sponsor	Empirical Status	Research approach	Data Type	Data Collection Method	Data Source	Key outcomes or concepts	
**Theme 1: Perceptions and experiences of clinical trials in RRR areas**														
Bearsley-Smith (28)	2008	VIC	RRR Only	Hospital and/or health service	Mental Health	No specific clinical trial explored		Empirical	Qual	Prim	Focus Groups	Clinicians	**Clinician perceptions** of clinical trials	★★⋆
Cusick (29)	2008	QLD	RRR Only	Hospital and/or health service	Rehabilitation and allied health	Clinical practice improvement	Non-com	Empirical	Quant	Sec	Document analysis	Clinician researcher diary	**Clinician experience** conducting a clinical trial	★★★
Francis (30)	2008	National	RRR Only	Specialty Society	Oncology	No specific clinical trial explored		Empirical	Quant	Prim	Survey	Advanced trainees in Oncology	Incentives for oncology trainee workforce retention	★★★
Sabesan (31)	2011	QLD	RRR Only	Hospital and/or health service	Oncology	No specific clinical trial explored		Empirical	Quant	Prim	Survey	Oncology patients	**Patient knowledge and attitudes** to clinical trials	★★★
Lee (32)	2022	VIC	RRR + Metro	Hospital and/or health service	Oncology	Drug (Phase 3)	Non-com	Empirical	Mixed	Prim	Survey Interview	Clinical trial participants	**Participant experience** with a clinical trial	★★★
Pye (33)	2023	NSW	RRR Only	Hospital and/or health service	Oncology	Drug (Phase 3)	Non-com	Empirical	Qual	Prim	Interviews	Oncology patients Trial participants	**Patient experience** of tele-oncology and teletrials	★★★
**Theme 2: Clinical trial capability and activity in RRR areas**														
Currow (34)	2006	SA	RRR + Metro	Primary Care (Palliative Care)	Palliative Care	Clinical practice improvement	Non-com	Non-empirical	n/a	n/a	n/a	n/a	**Implications and impact of RCT on health service delivery**	Not appraised
Heal (35)	2008	QLD	RRR	Primary Care (General Practice)	Wound Care	Treatment & procedures	Non-com	Non-empirical	n/a	n/a	n/a	n/a	Lessons **conducting a trial in primary care setting**	Not appraised
Delima (36)	2020	NT	RRR	Hospital and/or health service	Emergency medicine	Clinical practice improvement	Non-com	Non-empirical	n/a	n/a	n/a	n/a	**Impact of staff education** on trial recruitment	Not appraised
Sabesan (37)	2021	VIC, NSW, QLD	RRR + Metro	Hospital and/or health service	Oncology	Drug	Industry + Non-com	Non-empirical	n/a	n/a	n/a	n/a	Process and implementation of **the Australasian Teletrial Model**	Not appraised
Muthusamy (38)	2021	VIC	RRR	Hospital and/or health service	Oncology	Not specified	Industry + Non-com	Non-empirical	n/a	n/a	n/a	n/a	Review of the **Regional Trials Network Victoria program**	Not appraised
Thomas (39)	2023	National	RRR + Metro	Network or Collaborative	Oncology	Non-specific	Non-com	Empirical	Mixed	Prim	Questionnaire Interviews	Clinical trial network group members	**Use of telehealth** within cancer trials	★★★
Woollett (40)	2023	VIC	RRR + Metro	Hospital and/or health service	Oncology	No specific clinical trial explored		Empirical	Mixed	Prim	Consultation Interview Surveys Site Assessments	Oncology centres Site staff Trial stakeholders	**Clinical trial capability** framework	★★★
Nguyen (41)	2023	National	RRR + Metro	Network or Collaborative	Oncology	Treatment and procedures	Non-com	Empirical	Quant	Sec	Document analysis/audit	Network protocols and other secondary sources	Characteristics and diversity of **Investigators**	★★★
McPhee (42)	2024	VIC	RRR + Metro	Hospital and/or health service	Oncology	Drug; Treatment and procedure	Industry + Non-com	Empirical	Quant	Sec	Data extraction	Clinical trial management scheme (CTMS)	**Clinical trial availability and characteristics** across rural site locations	★★★
**Theme 3: Clinical trial recruitment in RRR areas**														
Watson (43)	2024	VIC	RRR + Metro	Primary Care (Community health)	Mental Health (maternal)	Clinical Practice Improvement	Non-com	Non-empirical	n/a	n/a	n/a	n/a	**Demand driven recruitment scalability** of a cluster RCT in rural areas	Not appraised
Lockery (44)	2019	TAS, VIC, ACT, NSW, SA	RRR + Metro	Primary care (GP)	Chronic disease management (Prevention)	Drug (Phase 4)	Non-com	Empirical	Quant	Sec	Data extraction	Recruitment database	Factors that contribute to successful **recruitment**	★★★
Tew (45)	2023	VIC	RRR Only	Primary care (GP)	Chronic disease management (T2DM)	Device	Non-com	Empirical	Quant	Prim	Questionnaire	GP practice staff	Site factors relating to **recruitment** efficiency	★★★
Alexander (46)	2023	VIC	RRR Only	Hospital and/or Health Services	Oncology	Drug (phase 3)	Non-com	Empirical	Quant	Sec	Costs estimated based on travel distance.	Travel distances derived from Google maps. Other cost estimates for assumed resource use and productivity impacts derived from market rates.	Scenario analyses **estimated the financial impact** of implementing a **networked trial model** from the participants’ perspective	★★★
Luiten-Apirana (47)	2023	NSW	RRR Only	Hospital and/or health service	Oncology	Not specified	Non-com	Empirical	Mixed	Sec	Administrative data, Interviews	Clinical trial assistance administration system, Clinical trial participants	Impact of the **clinical trial assistance pilot** on participants	★★★
Greaves (48)	2024	QLD	RRR + Metro	Primary care (GP)	Infectious disease and public health (COVID)	No specific clinical trial explored		Empirical	Quant	Sec	Data extraction	Australians Together Health Initiative (ATHENA) COVID-19 registry	Proportion and **characteristics of participants** who consent to re-contact for the clinical trial	★★★
**Theme 4: Clinical trial considerations for First Nations Australians**														
Marley (49)	2014	WA	RRR Only	Primary care (AMS)	Behavioural health (smoking cessation)	Behavioural therapy	Non-com	Empirical	Qual	Prim + sec	Workshops Interviews Data extraction	Research investigators and staff Staff Diaries Meeting Minutes	**Lessons** from an RCT involving First Nations Australians in a very remote setting	★★★
Abbott (50)	2021	Two States and one Territory	RRR + Metro	Primary care (AMS)	Infectious disease and public health (Otitis Media)	Clinical practice improvement	Non-com	Empirical	Qual	Prim + sec	Interviews Data Extraction	Parents/Carers Research Officers Healthcare providers Advisory Group Members Meeting Minutes	Considerations critical to ethical, sustainable and valuable RCTs **First Nations Australians** **Site Capability**	★★★
Cunningham (51)	2021	National	RRR + Metro	Registry	Oncology	Drug (phase 2 & 3) and non-drug trials	Industry + Non-com	Empirical	Quant	Sec	Data extraction	Australian New Zealand Clinical Trial Registry	Factors that could systemically exclude **First Nations Australians** from RCTs	★★★

State or territory names: QLD, Queensland; NSW, New South Wales; ACT, Australian Capital Territory; VIC, Victoria; TAS, Tasmania; SA, South Australia; WA, Western Australia; NT, Northern Territory. National indicates the study included or referred to all or the majority of Australian states or territories.

Geographic Setting: RRR, Rural, Regional and Remote Only setting; RRR + Metro, Rural, Regional and Remote, and metropolitan setting.

Sponsor: Non-com, non-commercial.

Research approach: Qual, qualitative; Quant, quantitative; Mixed, mixed-methods.

Data Type: Prim, Primary, Sec, Secondary.

Quality Rating: one star represents the lowest quality score (0–13), two stars indicate a mid-range score (14–26), and three stars is the highest quality category (27–39).

### 3.2 Characteristics, quality and evidence map of included publications

Publications were grouped thematically by key outcomes or concepts and arranged chronologically. The methodologies reported in the empirical publications (*n* = 18) were diverse, with nine quantitative, five qualitative and four mixed-methods studies. Eight used primary only, two used primary and secondary, and eight used only secondary data sources. The QuADS quality score for these publications ranged from 18 to 39. Among the studies involving primary data collection from individuals (*n* = 10), sample sizes were small, ranging from as few as two participants (33) to 178 (31).

Of all included publications that reported trial sponsor type, most were non-commercially sponsored trials (*n* = 16). Among the publications that reported the clinical trial intervention type, drug trials were the most common (*n* = 7) followed by clinical practice improvement interventions (*n* = 5). However, almost a third (*n* = 8) did not specify a clinical trial intervention type.

Figure 2 presents a visual summary of the distributed evidence in clinical trials conducted in RRR Australia. Half (*n* = 12) of all included studies were of trials conducted exclusively in RRR settings. One-third (*n* = 8) of all studies were of trials located exclusively in Victoria, while notable geographic gaps were evident in the Northern Territory, South Australia, and Western Australia, with Tasmania represented in only one multi-site trial. Clinical trial activity was heavily concentrated in oncology and cancer care, which accounted for half (*n* = 13) of all studies. Of the total studies, 15 were published since 2020, and nearly three-quarters (*n* = 11) focused on oncology, indicating a recent strong focus on improving RRR access to oncology trials. In contrast, other clinical areas such as chronic disease management, emergency medicine, and infectious diseases were markedly under-represented. Additional gaps were observed in specialised care services, including mental health, palliative care, and rehabilitation, where most studies were more than a decade old. Similarly, limited research was identified in primary care and community health settings, despite their foundational role in rural service delivery. An online interactive visualisation of the empirical evidence is available for further exploration. Refer to Supplementary File 3 to access the link.

**Figure 2:**
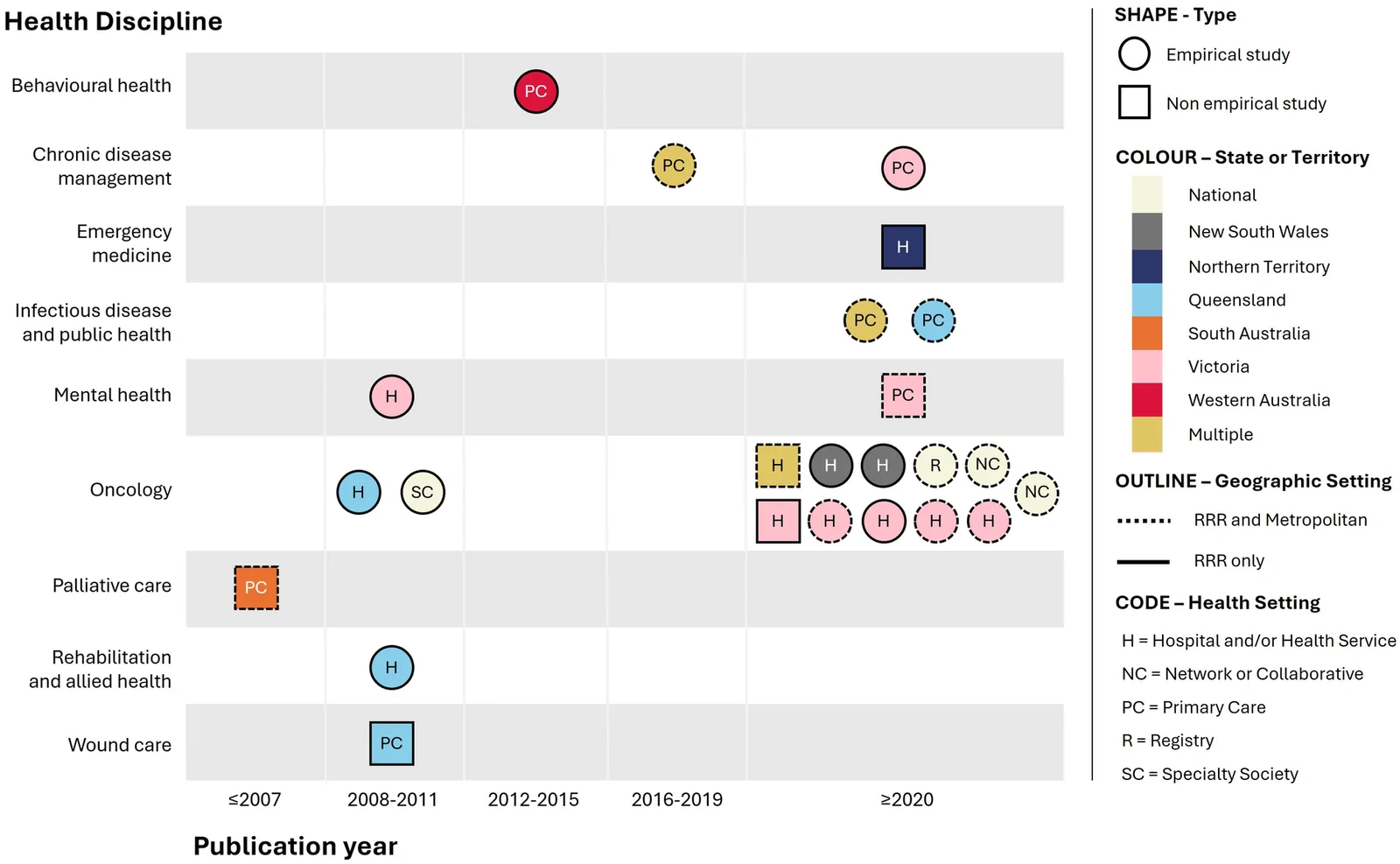
An evidence map of reported concepts relating to the conduct and development of clinical trial activity in RRR up to September 2024.

### 3.3 Synthesis of results

Four main themes arose from synthesising the key findings of each publication relevant to clinical trial activity and development in RRR Australia: 1) Perceptions and experience of clinical trials, 2) clinical trial activity and capability, 3) clinical trial recruitment, and 4) clinical trial considerations for First Nations Australians. Key outcomes from empirical studies are reported in Table 3.

**Table 3:** Thematic findings from empirical studies on clinical trial activity and development in rural, regional and remote Australia.

Theme	Description	Summary of relevant key findings of each publication
**1: Perceptions and experiences of clinical trials in RRR areas**	1. High appetite by RRR clinicians to conduct clinical trials. 2. Workforce issues and lack of clinician-researcher support systems were identified as barriers to conducting clinical trials. 3. Clinical trial access was considered important by patients, particularly oncology patients, but the cost and inconvenience of travel, and the need for a support network were the most important considerations in deciding whether to take part. 4. Telehealth models in clinical trials that alleviate the burden of travel were considered positive and acceptable in the absence of connectivity issues.	**Francis (2008)** (30)**:** 92% of Australian oncology advanced trainees surveyed felt access to clinical trials was an incentive for working in RRR areas. **Bearlsey-Smith (2008)** (28)**:** 17 Victorian RRR mental health clinicians felt conducting clinical trials benefited their practice, service and clients as well as a source of professional development, team and capacity building. Workforce issues, burden on the system and strict inclusion criteria were identified as barriers to conducting clinical trials in their setting. **Cusick (2008)** (29)**:** An emerging allied health clinician-researcher role in a remote Queensland setting was faced with few support systems (research leave, funding, training opportunities, mentorship, research infrastructure) and structural deterrents that were only overcome by persistence and personal investment of time and resources. **Sabesan (2011)** (31)**:** Of 178 RRR Queensland oncology participants, 56.3% said they would be willing to take part in a clinical trial (30.5% said unsure, 13.2% said not willing) with the top motivation being able to contribute to the advancement of medical research. Correct knowledge of clinical trials was significantly greater in patients living in regional areas (compared to rural and remote) and those with higher levels of education. Cost and inconvenience of travel and the need for personal support were considered most important considerations in taking part. **Lee (2022)** (32)**:** Of 23 regional Victorian oncology clinical trial participants interviewed, reported access to clinical trials was important and wanted, but time, cost, and need for personal support systems were barriers to accessing metropolitan-based clinical trials. The teletrial model (a form of decentralised clinical trial model) was considered acceptable, valued and equitable with decreased burden of travel, however, connectivity issues decreased the value of the service. **Pye (2023)** (33)**:** Of the seven rural New South Wales teleoncology participants interviewed, two took part in a teleoncology clinical trial. The acceptability of teleoncology was positive and was perceived to strengthen relationships through consistency of care, reduced the burden of travel and allowed participants to maintain independence and access to personal support networks.
**Theme 2: Clinical trial activity and capability in RRR areas**	1. Clinical trial activity, leadership and expertise has primarily been located in metropolitan areas. 2. There is a desire and opportunity to enhance diversity, reach and access to clinical trials by offering clinical trials closer to RRR Australians. 3. Different approaches have been explored to enhance clinical trial access for RRR patients and clinicians through telehealth models and modalities, and targeted health service capability building.	**Thomas (2023)** (39)**:** Involving members from Australian collaborative cancer trial groups who took part in a survey (*n* = 145) and interview (*n* = 21) found the employment of telehealth modalities in trials in response to COVID-19 was an opportunity to diversify, enhance reach and reduce the burden associated with travel by participants. Hybrid models (combination of face-to-face and telehealth) were the preferred remote model used in clinical trials. More is needed to overcome obstacles to the use of telehealth in trials, such as increased planning, engagement, capacity building, tailored technology and policy and guidelines. **Woollett (2023)** (40)**:** Involving six regional oncology centres in Victoria using integrative methods, six core factors were identified as critical for site development: trial infrastructure, leadership and culture, organisational support, technologies, staff skills, networks and collaborations. A clinical trial capability framework was developed that adopts a maturity model applied across all six core factors to support sites in assessing and developing their capability. **Nguyen (2023)** (41)**:** From exploring 120 investigator-led oncology trial protocols between 1989 and 2022, 100% of Chief Investigators and 84.9% of Principal Investigators were located in metropolitan areas, with 10.7%, 3.9% and 0% of Principal Investigators located in regional, rural and remote locations, respectively. **McPhee (2024)** (42)**:** Of 1669 clinical trials conducted across 43 clinical trial sites in Victoria in 2018, 98.9% were located in metropolitan and 10.1% in regional areas. Rural areas had a significantly lower proportion of phase 1, more phase 3, higher collaborative but fewer industry-sponsored trials than metropolitan sites. Rural sites had no clinical trials targeting head and neck, brain and CNS cancers and proportionately fewer trials for some cancers, and more for others.
**Theme 3: Clinical trial recruitment in RRR areas**	1. Suggestion that RRR clinical trial sites can be efficient and cost-effective, despite a small pool of potential participants. 2. RRR Australians are just as interested in clinical trials as their metropolitan counterparts. 3. Cost and travel are a known burden to clinical trial participation by RRR Australians recruited at metropolitan sites. This burden can be alleviated by the use of teletrial and telehealth models and travel cost assistance.	**Lockery (2019)** (44)**:** Regional GPs across five Australian states and territories were 45% more likely than GPs in metropolitan areas to recruit at least one randomised patient to a phase 3, investigator-led drug trial on aging. **Tew (2023)** (45)**:** Based on recruitment data for an investigator-led diabetes RCT, rural GP practices across Victoria were shown to be most efficient at recruiting clinical trial participants and have lower recruitment costs compared to GPs in metropolitan areas. Significance could not be determined due to the small sample size. **Alexander (2023)** (46)**:** The Victorian Networked Tele-Trial Model used for a phase 3 oncology trial in Victoria demonstrated a conservative mean cost (travel, time and productivity loss) difference for RRR participants of $2,155 for taking part locally using the model, opposed to travelling to a metropolitan centre. This cost difference was for the entire study (5 visits) for people living 119–325 km from the metropolitan study centre. **Luiten-Apirana (2023)** (47)**:** Of the 64 participants whose clinical trial travel and accommodation (mean cost of $1,704.65) was funded by the Clinical Trial Assistance Pilot in New South Wales, 27 took part in interviews and reported an alleviation of financial burden, reduced psychological distress and increased access to clinical trials as a result of the financial assistance. **Greaves (2024)** (48)**:** There was no difference in the proportion of COVID-19 patients living in RRR and metropolitan Queensland in consenting to be contacted to take part in a clinical trial. Both groups of patients were equally interested in participating in clinical trials if one was available to them.
**Theme 4: Clinical trial considerations for First Nations Australians in RR areas**	1) Clinical trial designs need to be more flexible regarding site location and exclusion criteria to avoid marginalising First Nations Australians. 2) Clinical trials involving First Nations Australians need to be highly adaptive, integrated into existing care, knowledgeable of communities, and positioned where local people can have ownership and involvement. 3) Funding models should consider cultural factors that might impact the cost and study milestone timelines.	**Marley (2014)** (49)**:** Standard clinical trial designs and funding guidelines do not consider the innate challenges and opportunities brought by conducting clinical trials in very remote Aboriginal Community Controlled Health Services in Western Australia. Trials were more expensive the more remote the site; required more staff to account for high staff turnover and absences; and were difficult to reach recruitment targets. Successful trials require community ownership, genuine relationship building and awareness of local cultural issues by the research team; clinical trials that address common conditions and where conduct can be embedded into standard practice; consideration of appropriate resourcing in anticipation of cultural events/business that might affect timelines. **Abbott (2021)** (50)**:** Key themes to ethical, sustainable and valuable clinical trials within 12 rural and metropolitan Australian Aboriginal Medical Services (AMS) involving 62 AMS stakeholders were identified: trial leadership by Aboriginal and Torres Strait Islander staff; provision of support to maintain AMS service delivery (cost and efficiencies); build and maintain relationship with AMS from beginning and beyond trial, and AMS workforce development and bidirectional capacity building. Differences between rural and metropolitan responses were not explored. **Cunningham (2021)** (51)**:** An analysis of 365 phase 3/2/NA oncology trials registered on the Australian and New Zealand Clinical Trial Registry between 2014 and 2019 found clinical trials that might exclude First Nation's Australians are those that had common exclusion criteria (multiple comorbidities or inadequate organ function, investigator judgement, ambiguous definition of psychological issues or history of substance abuse) and mismatch between location of trials (71% located in metropolitan areas) and types of cancers study that did not meet the needs of First Nations Australians.

#### 3.3.1 Theme 1: perceptions and experiences of clinical trials in RRR areas

Six empirical publications (28–33) were categorised under this theme and were published between 2008 and 2023. Three conducted before 2010 (28–30) and focused on clinicians’ perceptions and experiences of conducting clinical trials in RRR areas. In contrast, the three more recent studies (31–33) shifted focus to participants’ perceptions and experiences.

##### 3.3.1.1 Clinician perspectives

All publications that focused on clinicians’ perspectives (28–30) reported that clinical trials were perceived as positive and important for the clinician's career development. Notably Francis (30) found that 92% of oncology trainees surveyed indicated that improved access to clinical trials was an incentive for working in RRR areas.

##### 3.3.1.2 Participant perspectives

Publications that focused on patients’ perceptions and experiences in oncology clinical trials indicated that travel, time, distance and loss of support networks were the main perceived burdens to participating in metropolitan clinical trials by people living in RRR areas (31–33). People in rural and remote areas perceived this burden as more significant than those in regional areas (31). Publications by Lee (32) and Pye (33) explored participant acceptability of telehealth models (teletrials and clinical trials using teleoncology) for conducting clinical trials. Both publications reported participant acceptability was positive, with telehealth considered valuable and equitable due to the reduced travel burden and the ability for participants to retain a sense of independence.

#### 3.3.2 Theme 2: clinical trial activity and capability in RRR areas

Nine publications (34–42) were categorised under this theme, with all five non-empirical publications representing the earliest reported literature on this topic. Overwhelmingly, two-thirds of publications (*n* = 6) in this theme focused on the oncology health discipline.

##### 3.3.2.1 Research culture and workforce development

Research culture and workforce considerations were reported as both barriers and opportunities in regional settings. While Currow (34) and Heal (35) reported tensions between the demands of clinical trial rigour and the realities of resource-constrained regional health services, they also described benefits arising from trial implementation, including care redesign, relationship building, research infrastructure development, expanded workforce roles and education. Delima (36) reported that education delivered through a thiamine trial in Alice Springs increased awareness of Wernicke-Korsakoff syndrome and encouraged earlier thiamine administration beyond the trial itself.

##### 3.3.2.2 Clinical trial leadership

Clinical trial activity and leadership have historically been concentrated in metropolitan areas. One study found that all Chief Investigators and nearly 85% of Principal Investigators in oncology trials were based in metropolitan centres (41). Similarly, 98.9% of Victorian oncology trial sites in 2018 were metropolitan, with earlier phase and high-risk/complex trials rarely conducted in an RRR setting (42). As a consequence, 75.4% of rural-dwelling participants were enrolled in a clinical trial at a metropolitan site (42). Heal (35) argues that involving regional clinicians as co-investigators, providing research support, and allowing ‘greater ownership of the research agenda’ is needed to achieve clinical trial success in these regions.

##### 3.3.2.3 Telehealth and clinical trials

Opportunities were emerging to enhance reach and access to clinical trials with telehealth technology, improving clinical trial access and reducing travel burden for people in RRR Australia. Thomas (39) captured the early experience of telehealth use in clinical trials, instigated by COVID-19. In this study, trialists reported greater willingness to use telehealth in clinical trials involving a behavioural intervention or during follow-up phases of trials. This study recommended formalised telehealth capability-building through standardised processes, training and collaboration. Before COVID, however, Sabesan (37) had already conceptualised tele-oncology and went on to pilot the Australian Teletrial Model in 2018, a system-level telehealth approach using a hub-and-spoke supervision model between a primary and a satellite site, designed to overcome challenges of running a clinical trial when sites were not fully equipped.

##### 3.3.2.4 Clinical trial capability building initiatives

Three publications reported and described early initiatives to build clinical trial capability in Australian RRR health services (37, 38, 40). Sabesan (37) highlighted key opportunities from the piloted Australasian Teletrial Model, including collaborative effort, training, centralised support and incentives to address barriers to telehealth use in clinical trials. Additionally, Woollett (40) developed a clinical trial capability framework to build clinical trial capability in regional areas and enhance clinical trial access. This framework included six core factors required for a successful, independent clinical trial unit. A maturity model enabled Victorian health services to self-assess and guide their development of the core factors. Similarly, the Regional Trials Network Victoria was shown to increase the number of regional trials and participants by 63% and 49%, respectively, over two years by providing coordinated clinical trial support in a networked approach (38).

#### 3.3.3 Theme 3: clinical trial recruitment in RRR areas

Six publications in this theme reported outcomes related to clinical trial participant recruitment. Four publications (43–45, 48) were of trials conducted in primary care and two (46, 47) in hospital settings.

##### 3.3.3.1 Interest in clinical trials

Greaves (48) reported that 75% of 995 Queenslanders with COVID-19 consented to future clinical trial contact with no difference found between RRR and metropolitan patients. Watson (43) found there was such high demand and interest from RRR communities to participate in their cluster clinical trial that the study design was amended to include additional RRR clusters. This study found that achieving and maintaining an adequate sample size and power was feasible despite the additional costs required to support the additional RRR sites (43).

##### 3.3.3.2 Participant recruitment and retention

Two studies (44, 45) demonstrated that rural and regional general practices and practitioners were more efficient at recruiting clinical trial participants compared to metropolitan general practices for the respective clinical trials. Both studies (44, 45) supported the benefits of combining RRR and metropolitan recruitment sites to reduce the risk of trials being underpowered due to smaller recruitment pools at RRR sites.

##### 3.3.3.3 Financial considerations

In the hospital setting, Alexander (46) demonstrated a mean cost saving of $2155 per participant when attending a local site as part of a Networked Teletrial Model pilot, compared to the actual costs of travelling to a metropolitan centre for trial participation. Luiten-Apirana (47) demonstrated that for RRR participants who did need to travel to take part in a clinical trial, the financial support for travel and accommodation provided by NSW's Clinical Trial Assistance Pilot alleviated the financial burden, reduced psychological distress and increased access to clinical trials.

#### 3.3.4 Theme 4: Clinical trial considerations for First Nations Australians in RRR areas

Three empirical studies were categorised under this theme (49–51).

##### 3.3.4.1 Systemic exclusion

Cunningham and Garvey (51) found that First Nations Australians are systematically excluded from oncology clinical trial participation. This was due to: i) the location of trials in proximity to First Nations Australians (71% of trial locations explored were based in metropolitan regions); ii) types of cancers studied compared to the most common types of cancers affecting First Nations Australians; and iii) eligibility criteria more likely to exclude First Nations Australians (such as those based on comorbidities, ambiguous criteria such as psychological issues or history of substance abuse and investigator judgement).

##### 3.3.4.2 Ownership and responsiveness

Two publications (49, 50) emphasised the importance of First Nations ownership of research that is predominantly led and conducted by First Nations investigators and research staff. Marley (49) highlighted the need for research targeting First Nations people in Australia to be highly adaptive, integrated into existing healthcare, and informed by community knowledge. They also highlighted that standard clinical trial practices and funding models have undermined community control of clinical trials due to restricted budgets and time frames that do not allow for cultural responsiveness and reflexivity (49).

## 4 Discussion

### 4.1 Summary of evidence

This scoping review explored the academic literature for experiences, lessons and efforts to conduct and develop clinical trials in RRR Australia prior to the completion of the clinical trial capability initiatives funded by the Medical Research Future Fund 2019 RRR Clinical Trial Enabling Infrastructure Grant. This scoping review identified 24 publications that explored clinical trial activity, capability and growth in RRR Australia published up to and including September 2024.

A striking finding of this review is the concentration of clinical trial capability building efforts in oncology, particularly among studies published since 2020. While this may reflect the disease burden, funding priorities, commercial interest and the complex biology of cancer, a pattern recognised nationally (52) and globally (53), it has left substantial gaps in other areas of health need, including chronic disease, mental health, infectious disease, emergency care, rehabilitation and palliative care (1). Primary care and community health settings, which are central to healthcare delivery in many communities (54) were markedly under-represented. The reason for this discrepancy is unclear. It is uncertain whether it reflects lower levels of clinical trial activity in these disciplines and settings, or whether implementation experiences are occurring but not formally evaluated or reported. Consequently, the transferability of current knowledge regarding clinical trial capability building in RRR settings may be limited as the evidence base is disproportionately informed by oncology contexts.

Despite the small number of publications, several consistent themes emerged regarding clinical trial conduct in RRR Australia, many of which align with findings from stakeholder interviews conducted by our group (12). Access to clinical trials was perceived as important, particularly for oncology patients, but was constrained by workforce shortages, limited support for rural clinician trialists, and the travel costs and distances often required for patients to attend metropolitan study centres. Senate inquiries into rare cancers have similarly identified the lack of travel subsidy for clinical trial participation as a significant barrier for people living in RRR Australia, leading to recommendations that state and territory governments consider including clinical trials as part of current patient travel subsidy schemes (55, 56). However, only limited support is currently available in some jurisdictions (57) (58). Alongside travel assistance, strategies to improve local access are increasingly focused on strengthening rural trial capability through workforce training (59), decentralised trial models, telehealth, teletrials and remote monitoring (60).

Importantly, this review found evidence that people living in RRR areas are willing to participate in clinical trials (31, 43, 48) with several studies reporting that their recruitment and retention outcomes were comparable to those in metropolitan settings (44, 45). While smaller sample sizes can present challenges for trial feasibility and statistical power, authors emphasised that careful site selection and sampling strategies can support trial design (34, 43, 61). Several studies reported the need for mentorship and academic support for clinician trialist (29, 35) which has been reported elsewhere. Walsh (62) advocates for developing a place-based rural health research workforce that leverages local expertise to empower academics and clinicians in conducting community-focused clinical trials. This is supported by Jordan and colleagues (2025), who identified key enablers for rural participation in research (not specifically clinical trials); community engagement, benefit to the community, and involvement of trusted local health professionals (63). Strengthening local capability may not only improve access to trials but also enhance their relevance and sustainability within RRR communities.

A further theme was the importance of Indigenous leadership and culturally responsive trial design in research involving First Nations Australians. Studies included in this review highlighted the value of involving First Nations investigators, building trusted partnerships, and adapting trials to local cultural contexts and priorities. International evidence similarly suggests that trials involving Indigenous peoples are more likely to succeed when grounded in Indigenous knowledge systems, prioritised trust and partnership building, and incorporated culturally appropriate interventions (64). Together, these findings indicate that greater attention is need to instill Indigenous leadership, cultural governance and community partnership throughout the research process. While Australia has established guidelines and standards for ethical and responsible research with Aboriginal and Torres Strait Islander peoples, this review's findings indicate that further efforts are needed to strengthen implementation in practice and maximise the relevance and benefit of clinical trials for First Nations communities.

Consistent with global efforts to improve equitable access to clinical trials (65), several Australian initiatives have continued or emerged since 2021 to drive the agenda on equity of access to clinical trials for people living in RRR Australia. In Victoria, the Victorian Teletrial Collaborative aimed to “*optimise teletrial implementation within the cancer sector in Victoria”* was formed in 2021 (66), involving four funded teletrial initiatives operating in Victoria (Victoria Comprehensive Cancer Centre Alliance, Regional Trials Network-Victora, Alfred Health TrialHub and Safer Care Victoria). Nationally funded programs: 1. the Australian Teletrial Program, 2. The Rural, Regional and Remote (R3) clinical trial enabling program and 3. Regional Victorian Trials Alliance: Linkages, Innovation, Special populations, Equity, all funded between 2021 and 2026 by the MRFF 2019 RRR Clinical Trial Enabling Infrastructure Grant, are underway. The PARTNER network was established as part of the Australian Teletrial Program to connect RRR primary care practices to clinical trials. These initiatives aim to expand access to clinical trials across RRR Australia (67). Two of these programs [The Australian Teletrial Program (including PARTNER) and R3 Clinical Trial Enabling Program] are not specific to oncology or Victoria and indicate a growth of clinical trial capability across different health disciplines and jurisdictions in RRR Australia. Although these programs use innovative strategies to address the challenges of conducting trials across the RRR context, broader national reforms in health and medical research, led by the Intergovernmental Policy Reform Group, likely influenced their impact. These reforms include the National Clinical Trial Governance Framework [a framework consisting of hospital governance standards to conduct quality and safe clinical trials in all health services in Australia that choose to conduct trials (68)], Human Research Ethics accreditation, the National One-Stop-Shop (to harmonise clinical trial regulatory and approval processes across Australia) and National Standard Procedures (69). While not specifically designed to target RRR clinical trial capability, these reforms provide a robust foundation for clinical trial standards that all health services can build upon.

### 4.2 Strengths and limitations

This is the first scoping review and evidence map related to clinical trial activity in RRR Australia. As such, it offers important insights into the current state of the evidence and highlights critical areas for development. Moreover, the findings can inform the prioritisation of future clinical trial activity to better align with the needs of RRR communities.

A primary limitation is the small number of investigator-led empirical research publications across the four main thematic areas. This may reflect the review's criteria, which required studies to explicitly report on clinical trial activity, development, implementation, or lessons learned. As a result, the review's findings are limited in generalisability and breadth. This is not surprising, given that evaluating the implementation of clinical trials using research methodologies alongside the conduct of clinical trials is resource-intensive and often requires dedicated funding, planning and research capacity (19). Within each thematic area, the low number of empirical studies further highlights the need for more methodologically robust research on clinical trial implementation in the RRR context. This review shows that efforts to increase clinical trial access in RRR Australia are relatively recent, with the earliest empirical article published in 2008 and most published within the past decade. As most of these studies were conducted in Victoria and focused predominantly on oncology, more research is needed to understand and explore clinical trial concepts outside these contexts. Expanding this research agenda is essential to ensure that all Australians living in RRR areas can benefit equitably from participation in clinical trials that are relevant, accessible, and responsive to healthcare needs.

The review team did not involve a First Nations Australian in the review process. We acknowledge their leadership and engagement would have strengthened the review, particularly as this review focuses on RRR Australia, where First Nations Australians are prominent and where cultural awareness and safety are fundamental to all research being conducted in these areas.

### 4.3 Conclusion

Clinical trial access by people living in RRR Australia is considered important, but efforts to achieve this have been inadequate. Before the Australian Government's investment into enabling RRR clinical trial infrastructure efforts to increase access from travel subsidies to initiatives aimed to bring trials closer to home have been considered, however, they are at the very initial stages of maturity. Historically, clinical trials have been challenging to conduct in RRR areas, despite the appetite to develop decentralised trials. The greatest efforts to enhance access to date are for oncology trials, particularly in Victoria since 2020. First Nations Australians are shown to be systematically excluded from trials, and improved access requires not only bringing trials closer to home for them, but also a concerted effort to design trials that are meaningful and beneficial, and that consider the historical and cultural contexts of the community in which the trial will be conducted. There is evidence of momentum continuing to build, and we highly recommend more research that assesses the impact, experiences and processes from implementing such clinical trial initiatives so that lessons can be rapidly taken up by others working in this space. We also recommend that in addition to the innovative models being trialled to conduct clinical trials in RRR areas, a place-based approach be considered in each health service or site to strategically develop a clinical trial workforce, capability and capacity that benefits the community it serves.

## Data Availability

The original contributions presented in the study are included in the article/Supplementary Material, further inquiries can be directed to the corresponding author.
